# A bibliometric study and visualization analysis of the current status and perspectives in upper tract urothelial carcinoma

**DOI:** 10.1097/MD.0000000000048126

**Published:** 2026-03-27

**Authors:** Shuying Wang, Sheng Chen, Xiaohan Ma, Tong Wu, Peiling Zuo, Xin Tang, Yan Zhang, Xiaofei Zhang, Encun Hou

**Affiliations:** a Graduate School, Guangxi University of Chinese Medicine, Nanning, Guangxi, China; b Ruikang Hospital, Guangxi University of Chinese Medicine, Nanning, Guangxi, China.

**Keywords:** bibliometrics, cancer, citespace, upper tract urothelial carcinoma, UTUC, VOSviewer

## Abstract

**Background:**

Upper tract urothelial carcinoma (UTUC) is a rare and highly aggressive malignancy primarily occurring in the upper urinary tract, including the renal pelvis and ureter. Despite recent advancements in treatment strategies, the diagnosis and management of this disease remain challenging. This study proposes a systematic bibliometric evaluation of collaborative networks and scholarly impact across national, institutional, individual author, and journal levels within UTUC research. The goal is to evaluate the evolution of knowledge structure clusters and identify trending topics and emerging issues.

**Methods:**

A topic-based search strategy was employed to retrieve articles and reviews related to UTUC from the Web of Science core database bibliometric analysis was conducted using Citespace and VOSviewer.

**Results:**

This investigation analyzed 3621 publications spanning 83 countries, where American and Japanese researchers demonstrated the highest productivity. Publications related to UTUC have been increasing year by year. The primary research institutions include the Medical University of Vienna, UT MD Anderson Cancer Center, Kaosiung Medical University, and Université de Montréal. Publication analysis reveals “UROL ONCOL-SEMIN ORI” as the predominant outlet for article dissemination in this field, while citation networks identify “Journal of Urology” as the most influential reference source. The author network encompasses 14,918 researchers, among whom Shariat SF, Rouprêt M, Margulis V, and Lotan Y have shown exceptional productivity, with Rouprêt M achieving particular prominence through co-citation frequency. Through co-citation analysis, a macro sketch and micro characterization of the entire knowledge domain are achieved. Current and developing research areas include FGFR3 mutations, molecular immunotherapy targeting, and tumor localization. “Pembrolizumab,” “multicenter,” “survival,” and “transitional-cell carcinoma” may also represent new trends and focal points for future research.

**Conclusion:**

The analysis reveals a clear trajectory toward more rigorous scientific inquiry in UTUC investigations, with particular emphasis on developing standardized protocols for tumor localization, surgical approaches, and novel treatment modalities. This evolving paradigm reflects the discipline’s dedication to advancing therapeutic precision while minimizing risks, suggesting significant potential for subsequent research to refine clinical decision-making and improve prognostic results.

## 1. Introduction

Upper tract urothelial carcinoma (UTUC) is a malignant tumor originating from the epithelium of the renal pelvis or ureter.^[1,2]^ It accounts for 5% to 10% of all urothelial carcinomas and is a relatively rare urological tumor.^[3,4]^ Compared to the more common bladder urothelial carcinoma, UTUC tends to be more aggressive, with early symptoms being subtle and often progressing to an advanced stage at diagnosis.^[5]^ UTUC’s unique anatomical location, concealed early symptoms, high invasiveness, and metastatic potential contribute to a high risk of postsurgical recurrence.^[6]^ Consequently, this malignancy exhibits distinct biological behavior and poses unique clinical challenges. Compared to bladder cancer, UTUC is typically diagnosed at a more advanced stage, with over 60% of patients presenting with invasive disease at initial diagnosis, which generally results in a poorer prognosis.^[7,8]^

Over the past few decades, the diagnostic and therapeutic approaches for UTUC have undergone significant evolution. Conventional diagnostic methods, such as urine cytology and computed tomography urography, exhibit limitations in sensitivity and specificity. Although ureteroscopy (URS) combined with biopsy represents the diagnostic gold standard, it is an invasive procedure. In terms of treatment, radical nephroureterectomy (RNU) has long been regarded as the standard treatment plan for high-risk or muscle-invasive UTUC. However, this procedure results in the loss of renal function on the affected side, posing significant challenges for patients with insufficient renal functional reserve or a solitary kidney.^[9]^ Consequently, kidney-sparing surgery has been increasingly adopted for low-risk UTUC patients, aiming to balance tumor control with preservation of renal function.^[10]^

In recent years, with the advancement of molecular biology research and the rise of precision medicine, the field of UTUC research has entered a period of rapid development. Particularly in systemic therapy for advanced or metastatic UTUC, breakthrough progress has been achieved. Novel agents represented by fibroblast growth factor receptor (FGFR) inhibitors (e.g., erdafitinib),^[11]^ immune checkpoint blockers (e.g., pembrolizumab),^[12]^ and antibody–drug conjugates (e.g., enfortumab vedotin)^[13]^ have significantly transformed the treatment landscape for advanced urothelial carcinoma, offering new hope for patients with UTUC. These advances have not only driven changes in clinical practice but have also spurred the publication of numerous related basic and clinical studies.

With the exponential growth of literature related to UTUC, researchers and clinicians face the challenge of comprehensively and promptly grasping the research status, hotspots, frontiers, and future trends in this field. Although traditional literature reviews can provide in-depth insights, they are often influenced by the authors’ subjectivity and struggle to quantitatively and objectively reveal the structure and evolution of the entire knowledge domain from a macro perspective.^[14]^ Bibliometrics, as a scientific method that employs mathematical and statistical techniques to quantitatively analyze literature in a specific field, can effectively compensate for the limitations of traditional reviews. Through the visual analysis of data such as authors, institutions, countries, keywords, and citations, it clearly presents the knowledge map of a research area, identifies core research forces, untangles research hotspots, and predicts future development directions.^[15]^

Therefore, this study aims to perform a comprehensive and systematic bibliometric analysis of the global research literature on UTUC over the past 25 years. Through this analysis, we delineate the overall developmental trajectory of UTUC research, identify the most influential countries, institutions, and scholars, elucidate key research hotspots and evolutionary pathways, and provide valuable references and directions for future scientific investigations and clinical practice.

## 2. Materials and methods

### 2.1. Database

The Web of Science Core Collection (WoSCC) is widely recognized as the foremost and extensive database in scientific research, encompassing a vast array of academic literature and studies.^[16]^ In the domain of scientometric research, the Web of Science Core Collection has established itself as the benchmark database, offering unparalleled data richness and formatting precision that seamlessly interfaces with specialized literature analysis platforms. In this study, we conducted an exhaustive literature search using WoSCC.

### 2.2. Search strategies

On February 1st, 2025, to ensure data consistency and minimize temporal fluctuations in database records, we extracted all relevant publications spanning 2000 to 2025 from the Web of Science Core Collection. The search strategy incorporated the following key terms: TOPIC = (upper urothelial carcinoma) OR (upper urinary tract urothelial carcinoma) OR (urothelial carcinoma of the upper urinary tract) OR (UTUC) OR (upper tract urothelial carcinoma) OR (renal pelvic urothelial carcinoma) OR (ureteral urothelial carcinoma). To avoid bias from discrepancies, the retrieval was cut off on February 1st, 2025.

### 2.3. Data extraction and analysis

**Figure 1.**
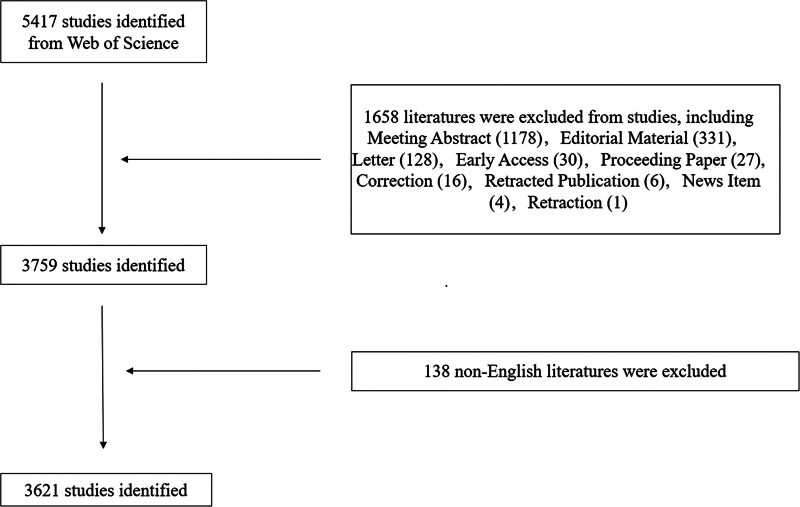
Comprehensive search procedure.

A total of 5417 articles related to UTUC were retrieved from the WoSCC database. Among them, there are 11 types of literature. As shown in Table 1, a total of 3181 articles, accounting for 57.73% of all papers, represent the most common category. The second most common type is the Review Article, with 578 pieces, accounting for 10.67% of the total. The remaining 9 types of literature are Meeting Abstract (1178), Editorial Material (331), Letter (128), Early Access (30), Proceeding Paper (27), Correction (16), Retracted Publication (6), News Item (4), and Retraction (1). Finally, we excluded 138 non-English articles. For this study, we ultimately selected original research, reviews and meta-analyses related to UTUC, resulting in a final analysis of 3621 articles. The detailed filtering process is illustrated in Figure 1.

**Table 1:** Document types of the publications.

Rank	Document types	Publications	% of 5417	Citations	H-Index
1	Articles	3181	58.73	66,446	102
2	Meeting Abstract	1178	21.74	212	5
3	Review Article	578	10.67	16,476	62
4	Editorial Material	331	6.11	436	9
5	Letter	128	2.36	315	7
6	Early Access	30	0.55	46	4
7	Proceeding Paper	27	0.50	1156	14
8	Correction	16	0.30	1	1
9	Retracted Publication	6	0.11	35	4
10	News Item	4	0.07	1	1
11	Retraction	1	0.02	0	0

### 2.4. Data analysis and visualization

Originally developed by researcher Chaomei Chen, this analytical platform facilitates the visualization and evaluation of scholarly patterns, including collaborative relationships, field-specific focus areas, conceptual frameworks, current trends, and future directions in scientific research.^[17]^ Our analytical methodology incorporated advanced scientometric software packages (CiteSpace version 6.2.R3 [Drexel University, Philadelphia] and VOSviewer [Centre for Science and Technology Studies {CWTS}, Leiden University, Leiden, The Netherlands]) to systematically investigate: multilevel cooperative relationships among research entities, citation-based intellectual connections between scholarly sources, macro-level knowledge domain representations through dual-map overlays. The CiteSpace configuration parameters were established as follows: temporal segmentation spanned January 2000 to February 2025 with annual intervals, textual analysis incorporated document titles, abstracts and author keywords, nodal categories were individually analyzed (including geographic, institutional, authorial, terminological, and co-citation elements), selection thresholds employed g-index values (*K* = 25 or 20) and network simplification utilized slice-wise pruning (all maintained at software-default settings).

VOSviewer (version 1.6.20) developed by Leiden University excels in creating, visualizing, and exploring maps based on network data.^[18]^ It plays a significant role in the visualization of countries, institutions and authors. The software facilitates generation of knowledge maps that visually represent relationships between research fronts and scholarly publications through multiple analytical dimensions. Within the VOSviewer environment, individual nodes correspond to distinct elements, where dimensional attributes (node size and hue variation) quantitatively represent frequency and category distribution. Internodal linkages are weighted to indicate strength of relational ties or co-citation frequency.

## 3. Results

### 3.1. Temporal distribution map of publications and citations

**Figure 2.**
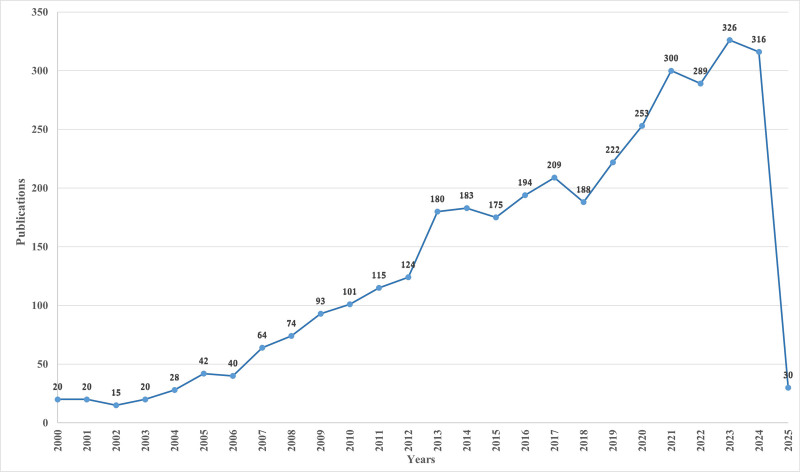
Annual publication volume trend from 2000 to 2025.

Firstly, changes in annual publications and citation frequencies reflect the speed, progress, and research focus of this study. A total of 3621 articles related to UTUC were published between 2000 and 2025, demonstrating an overall increasing trend. The number of published papers on this topic increased slowly from 2000 to 2005, decreased slightly in 2006, rose from 2007 to 2011, and showed a significant growth trend from 2012 to 2013, maintaining steady growth until 2017. Although there was a slight decline in publications from 2017 to 2018, academic output related to UTUC showed a rapid development trend from 2018 to 2024. By 2023, the number of annual publications in this field reached an unprecedented peak, which greatly reflects the scientific community’s deep exploration and widespread interestin this topic (Fig. 2).

### 3.2. Analysis of leading countries, region, and institution

**Figure 3.**
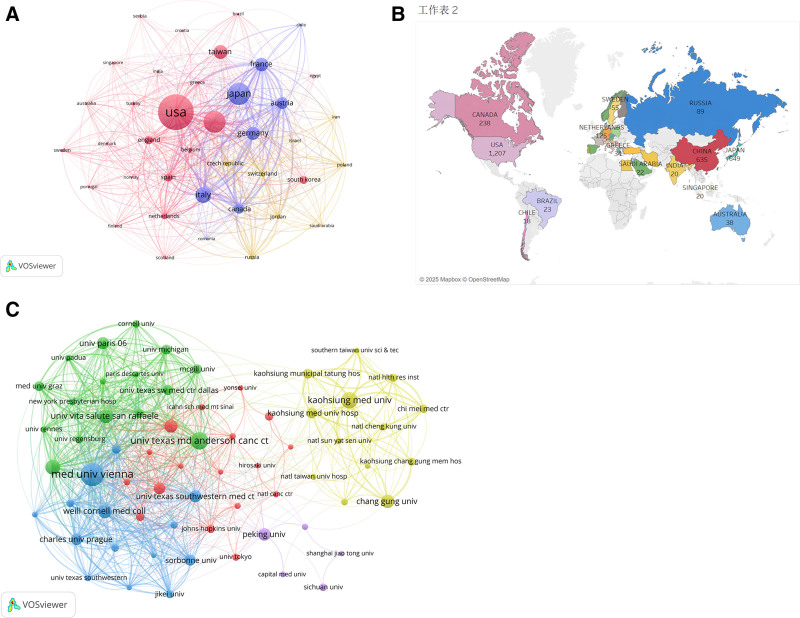
(A) The visualization of countries. (B) Country/region cooperation map on UTUC. (C) The network map of institution. UTUC = upper tract urothelial carcinoma.

By analyzing the collaboration network map in UTUC-related studies, we identified the top 10 contributing countries and regions (Table 2, Fig. 3A). Based on a criterion of having published at least 15 articles, we selected 39 countries for inclusion in our study. The heatmap (Fig. 3B) illustrates that these articles are primarily concentrated in North America, Asia, and Western European countries. Among them, the USA ranks first with 1207 articles, followed by Japan (n = 649), China (n = 635), Italy (n = 419), and Taiwan (n = 358). The observed publication distribution underscores both the international scope and geographic breadth of UTUC investigations, with substantial contributions emerging from multiple European and Asian nations. Network visualization parameters employ proportional circle sizing to denote national publication output and weighted connecting edges to represent collaborative frequency. This spatial analysis reveals significant regional disparities in UTUC research engagement. Notably, the most intensive collaborative networks involve 5 nations (United States, Italy, Austria, Germany, and France), suggesting an accelerating pattern of transnational research partnerships in urologic oncology. Additionally, we selected institutions that have published at least 30 articles, resulting in a total of 65 institutions included in the study. Table 3 shows that the Medical University of Vienna has published the most articles (n = 212), followed by UT MD Anderson Cancer Center (n = 145) and Kaosiung Medical University (n = 134). Most of the top 10 institutions are located in Western Europe and East Asian countries. By examining the strength of collaborative connections and the institutional network collaboration diagram (Fig. 3C), it can be observed that UT MD Anderson Cancer Center, Medical University of Vienna, Vita-Salute San Ranffaele University, and the University of Texas Southwestern Medical Center at Dallas display numerous intersecting lines, indicating more frequent collaboration among these institutions. Bibliometric evidence indicates substantial opportunities remain for expanding worldwide collaborative networks among research institutions conducting UTUC studies.

**Table 2:** The top 10 productive countries/region on the research of UTUC.

Rank	Countries/regions	Publications	Citations	Link strength	H-Index
1	USA	1207	41,188	2057	94
2	Japan	649	15,178	910	58
3	China	635	6253	335	33
4	Italy	419	13,880	1706	60
5	Taiwan	358	6397	98	35
6	France	341	15,883	1319	66
7	Germany	333	15,440	1383	66
8	Austria	296	13,711	1544	56
9	Canada	238	10,726	1154	57
10	England	182	10,069	606	50

UTUC = upper tract urothelial carcinoma.

**Table 3:** The top 10 productive institution on the research of UTUC.

Rank	Institutions	Publications	Citations	Countries
1	Medical University of Vienna	212	8429	Austria
2	UT MD Anderson Cancer Center	145	7798	USA
3	Kaosiung Medical University	134	2029	Taiwan
4	Université de Montréal	119	6505	Cannada
5	Weill Cornell Medicine	109	3550	USA
6	Vita-Salute San Ranffaele University	108	4829	Italy
7	Chang Gung University	102	1953	Taiwan
8	Keio University	100	5076	Japan
9	Peking University	96	1324	China
10	University of Texas, Southwestern Medical Center at Dallas	89	1558	USA

UTUC = upper tract urothelial carcinoma.

### 3.3. Analysis of leading journals

To identify the most productive and influential journals, we utilized VOSviewer software to visualize the published journals related to UTUC. Our journal selection criteria required a minimum publication threshold of 100 articles, yielding 148 qualified journals for evaluation. As detailed in Table 4, examination of 496 academic periodicals revealed ``UROL ONCOL-SEMIN ORI’’ as the most prolific (n = 193), ahead of ``BJU International’’ (n = 175), ``World Journal of Urology’’ (n = 156), and ``Journal of Urology’’ (n = 145). Notably, the top 10 included 1 Q1-ranked journal and 2 with Impact Factors exceeding 5.0. Applying a co-citation threshold of 150, we identified 112 journals for inclusion in our analysis. Examination of 5743 co-cited journals revealed ``Journal of Urology’’ as the most frequently reference (n = 12,894, IF = 6.40) followed by “European Urology” and “BJU International.” The majority (70%) of these journals were classified in Q2, Q3 JCR categories, with 2 top-10 journals boasting impact factors above 10.

**Table 4:** The top 10 productive and cited journals on the research of UTUC.

Rank	Sources	Counts	JCR	IF (2024)	Co-cited sources	Citations	JCR	IF (2024)
1	UROL ONCOL-SEMIN ORI	193	Q3	2.4	Journal of urology	12,894	Q2	6.4
2	BJU International	175	Q2	3.7	European urology	11,539	Q1	25.3
3	World Journal of Urology	156	Q2	2.8	BJU international	6232	Q1	3.7
4	Journal of Urology	145	Q2	6.4	Urology	5875	Q3	2.1
5	Urology	124	Q3	2.1	World journal of urology	3201	Q2	2.8
6	Frontiers in Oncology	99	Q3	3.5	Journal of clinical oncology	2884	Q1	42.1
7	International Journal of Urology	96	Q3	1.8	UROL ONCOL-SEMIN ORI	2720	Q3	2.4
8	Journal of Endourology	96	Q2	2.9	cancer-am cancer soc	2683	Q2	6.1
9	Clinical Genitourinary Cancer	92	Q3	2.3	Journal of endourology	2251	Q2	2.9
10	European Urology	81	Q1	25.3	International Journal of Urology	1656	Q3	1.8

UTUC = upper tract urothelial carcinoma.

**Figure 4.**
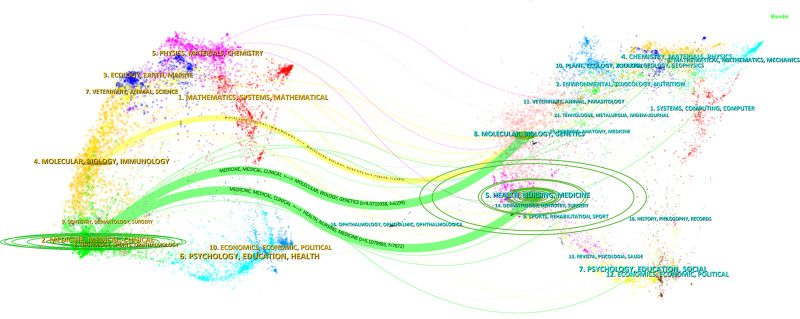
Dual-map overlay of UTUC research. UTUC = upper tract urothelial carcinoma.

The essence of double-map overlay lies in the connection between the citing domain and the cited domain. Through the overlay map, knowledge flows between disciplines at the journal level can be reflected.^[19]^ It illustrates the citation relationship between journals and co-cited journals, with the citing journal cluster on the left, the cited journal cluster on the right, and the curves representing citation connections, fully demonstrating the context of citations.^[20]^ The map (Fig. 4) reveals that citations related to UTUC primarily involve journals in Medicine, Medical and Clinical fields, with significant overlaps with Health, Nursing, Molecular, Biology, and Genetics, indicating the interdisciplinary nature of UTUC research.

### 3.4. Analysis of leading authors and co-cited authors

**Figure 5.**
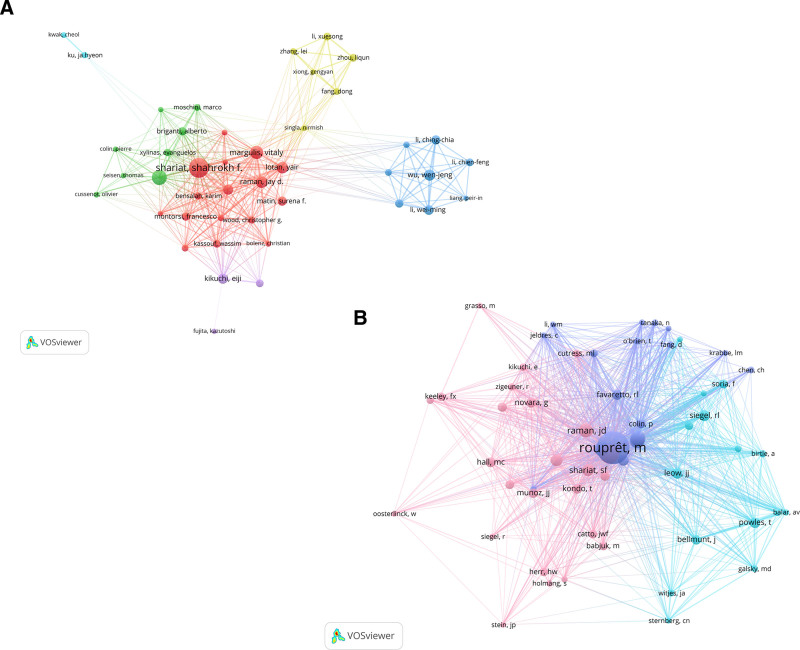
(A) Author collaboration chart for publication volume. (B) Co-cited author collaboration chart.

The author collaboration network visualization (Fig. 5A) utilizes chromatic encoding to distinguish 5 principal research clusters, with connecting edges quantitatively representing partnership intensity. This structural analysis reveals significant cooperative relationships existing among investigators. For this study, a criterion of having published at least 30 articles was established, resulting in the inclusion of 44 authors in the analysis. Table 5 lists the top 10 authors by the number of published papers, including Shariat, Shahrokh F. (n = 221), Roupret, Morgan (n = 143), Margulis, Vitaly (n = 114), Lotan, Yair (n = 98), and Raman, Jay D. (n = 95). Our analysis incorporated authors with ≥160 citations (n = 58). Co-citation mapping (Fig. 5B) highlights 5 influential scholars: Rouprêt M (n = 2101) leads, followed by Margulis V (n = 974), and others, confirming their dominant intellectual contributions to UTUC literature.

**Table 5:** The 10 top productive and cited authors on the research of UTUC.

Rank	Authors	Publications	Co-cited authors	Citations
1	Shariat, Shahrokh F.	221	Rouprêt, M	2101
2	Rouprêt, M	143	Margulis, V	974
3	Margulis, Vitally	114	Seisen, T	661
4	Lotan, Yair	98	Lughezzani, G	649
5	Raman, Jay D.	95	Xylinas, E	642
6	Wu, Wen-Jeng	89	Raman, JD	611
7	Karakiewicz, Pierre I.	79	Shariat, SF	472
8	Kikuchi, Eiji	79	Roscigno, M	471
9	Li, Wei-Ming	72	Siegel, RL	426
10	Matin, Surena F.	67	Rink, M	418

UTUC = upper tract urothelial carcinoma.

### 3.5. Analysis of keywords and burst words

**Figure 6.**
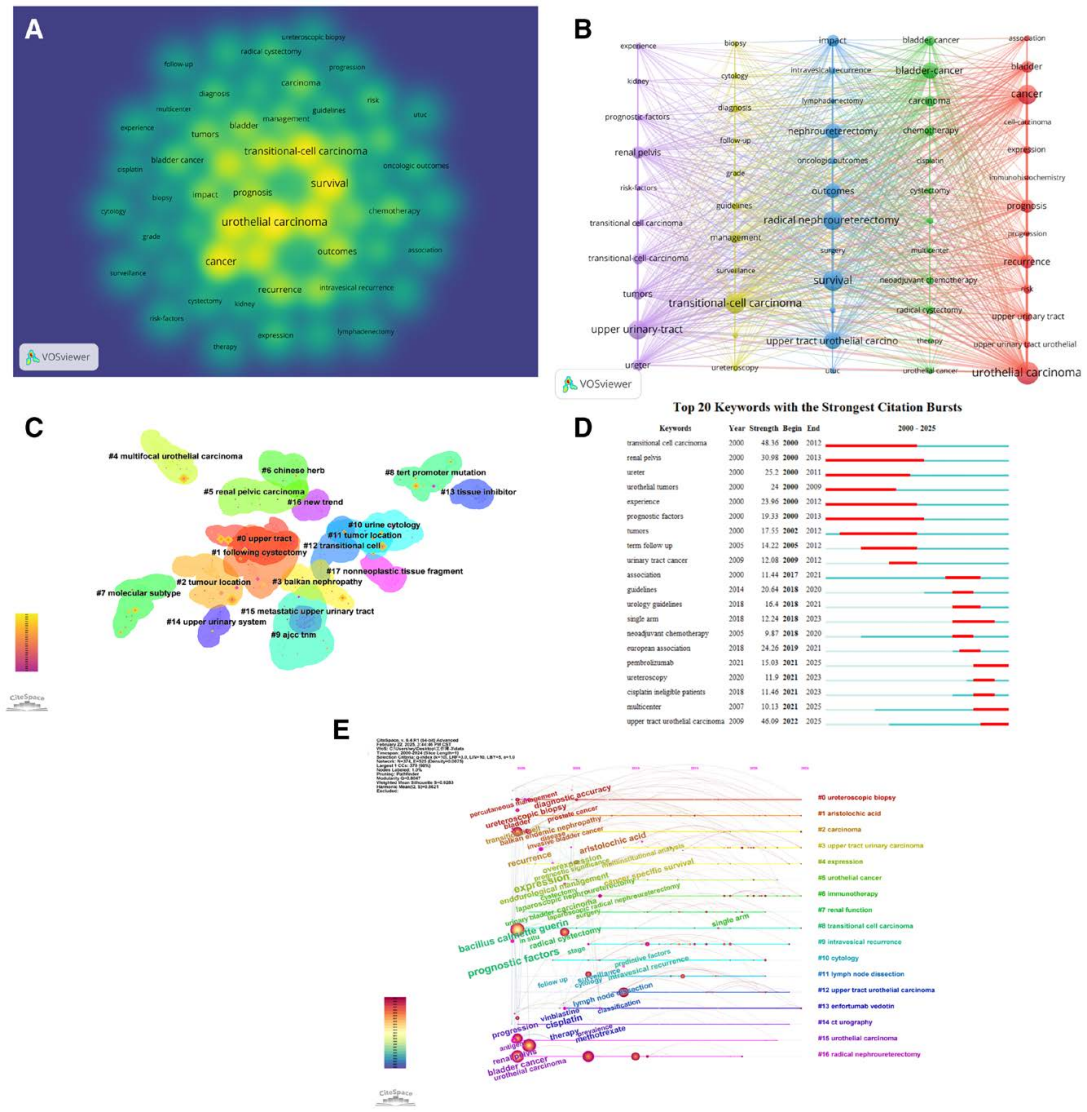
(A) Map of keywords in UTUC research. (B) Co-occurring map of keywords. (C) Thematic keyword cluster mapping. (D) Ranking of top 20 terms demonstrating citation bursts. (E) The temporal evolution of keyword clusters is illustrated in panel. UTUC = upper tract urothelial carcinoma.

Keywords represent standardized lexical units extracted from article titles and content to characterize research themes, serving as valuable tools for information categorization. In scientometric investigations, keyword analysis enables precise detection of emerging research frontiers and prevalent topics, establishing itself as a fundamental methodology in bibliographic studies.^[21]^ Supplementary to the primary search parameters, keyword analysis was conducted using VOSviewer on metadata elements (titles and abstracts) from the collected 3621 articles. From the 7189 identified keywords, quantitative evaluation demonstrated that 509 terms had a frequency exceeding 10 occurrences, while a subset of 123 terms appeared >50 times in the corpus. We retained only keywords that appeared 100 times, resulting in 58 keywords included in the study scope. As evident from Figure 6A and Table 6, “urothelial carcinoma” emerged as the most profound term, occurring 1076 times, followed by “survival” (n = 917), “transitional-cell carcinoma” (n = 875), “cancer” (n = 825), “radical nephroureterectomy” (n = 800), and “upper urinary-tract” (n = 800). The research hotspots encompassed areas such as urological tumors, cancer treatment, management, and prognostic survival, as illustrated in Figure 6B.

**Table 6:** The top 20 keywords.

Rank	Keyword	Occurrences	Link strength	Rank	Keyword	Occurrences	Link strength
1	Urothelial carcinoma	1076	6277	11	Recurrence	460	3411
2	Survival	917	6350	12	Prognosis	439	2889
3	Transitional-cell carcinoma	875	5794	13	Renal pelvis	406	2877
4	Cancer	825	4901	14	Bladder	394	2387
5	Radical nephroureterectomy	800	5588	15	Impact	373	2764
6	Upper urinary-tract	800	5441	16	Ureter	348	2439
7	Upper tract urothelial carcinoma	649	3745	17	Tumors	337	2091
8	Bladder-cancer	627	3736	18	Carcinoma	332	1920
9	Nephroureterectomy	562	3941	19	Transitional-cell-carcinoma	327	2104
10	Outcomes	525	3578	20	Chemotherapy	313	2150

There are 17 major clusters of burst detections (Fig. 6C), including “#0 upper tract,” “#1 following cystectomy,” “#2 tumor location,” “#3 Balkan nephropathy,” and “#4 upper urinary system,” among others. Each group is color-coded to represent a different area of research, allowing for easy identification of major trends and focus in the research.

Burst detection analysis identifies terminology showing statistically significant increases in citation frequency over defined time intervals, serving as indicators of emerging scientific trends.^[22,23]^ The visualization in Figure 6D ranks the 20 most prominent keyword bursts from 2000 to 2025, with red bars highlighting terms gaining substantial academic traction and green bars denoting declining concepts. Evidently, “transition cell carcinoma” emerges as the longest-cited keyword, while early keywords (2000–2013) are primarily focused on areas such as urology, cancer research, and prognostic factors. As time progresses, terms like “pembrolizumab,” “multicenter,” and “upper tract urothelial carcinoma” are likely to become focal points of research in the coming years.

This study employed CiteSpace for keyword timing analysis (Fig. 6E). The clustering results showed a modularization index *Q* = 0.8047 and a silhouette value *S* = 0.9283, indicating a significant structure. Before 2024, research focused on the diagnosis and treatment of urological tumors and chemotherapy drugs (such as prostate cancer, bladder cancer, cisplatin, etc). From 2024 to 2025, 3 major shifts emerged: the application of radiomics techniques (such as computed tomography [CT] urography) for early diagnosis of upper urinary tract carcinomas; the introduction of novel therapies (such as enfortumab vedotin combined with immunotherapy) into clinical practice; the concurrent advancement of minimally invasive surgeries (such as laparoscopic nephroureterectomy) and molecular marker research, marking the transition towards precision medicine in this field.

### 3.6. Co-cited references and reference with citation bursts

**Figure 7.**
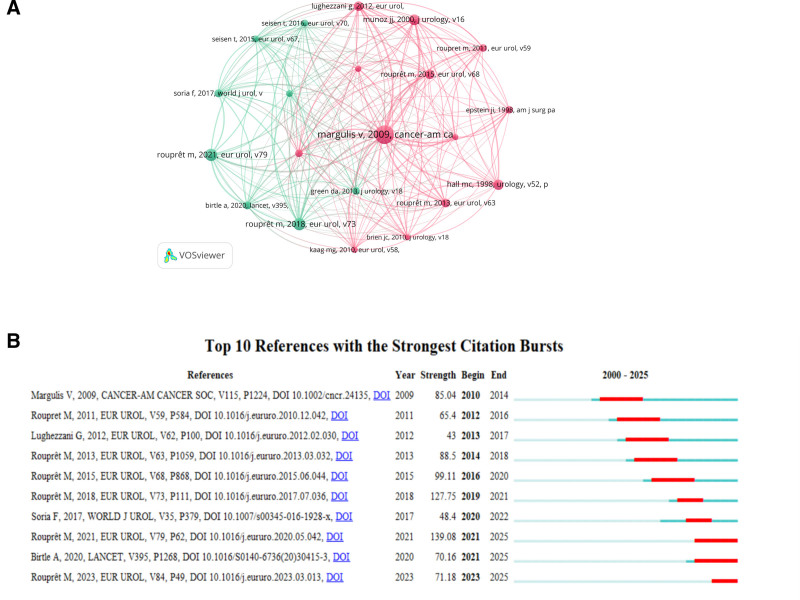
(A) Presents the network visualization of co-cited references in UTUC research. (B) The top 10 references with the strongest citation bursts. The vibrant red segments represent high-frequency citation periods, while green portions indicate lower citation activity. UTUC = upper tract urothelial carcinoma.

Over the past 2 decades, 47,325 articles related to UTUC research have been co-cited. In our selection process, we chose articles that had been cited >100 times, resulting in a total of 70 articles for analysis. The top 10 co-cited articles, listed in Table 7, have each received at least 224 co-citations. Using VOSviewer for co-citation analysis (Fig. 7A), we determined that Margulis V’s classic article “Outcomes of radical nephroureterectomy: a series from the Upper Tract Urothelial Carcinoma Collaboration,^[9]^” published in Cancer in 2009, has the highest co-citation intensity with 723 co-citations. This study emphasizes that radical nephroureterectomy provides durable local control and cancer-specific survival in patients with localized UTUC. It also identifies pathological tumor grade, T stage, lymph node status, tumor architecture, and lymphovascular invasion as important prognostic variables related to oncologic outcomes. These factors may aid in selecting patients suitable for adjuvant systemic therapy. Additionally, Rouprêt M’s article “European Association of Urology Guidelines on Upper Urinary Tract Urothelial Carcinoma: 2017 Update^[24]^” published in European Urology in 2018, ranks second. These guidelines provide a comprehensive update on the diagnosis and treatment of UTUC highlighting its rarity and aggressiveness. It notes that 60% of patients present with invasive tumors at diagnosis, emphasizing the importance of timely and appropriate diagnosis.

**Table 7:** The top 10 co-cited references.

Rank	Co-cited reference	Co-citation	Journals
1	Margulis V, 2009, Cancer: Am Cancer Soc, v115, p1224	723	Cancer
2	Rouprêt M, 2018, Eur Urol, v73, p111,	415	European Urology
3	Rouprêt M, 2021, Eur Urol, v79, p62,	410	European Urology
4	Munoz JJ, 2000, J Urology, v164, p1523	347	Journal of Urology
5	Hall MC, 1998, Urology, v52, p594,	323	Urology
6	Lughezzani G, 2012, Eur Urol, v62, p100	288	Science Direct
7	Rouprêt M, 2015, Eur Urol, v68, p868,	281	European Urology
8	Rouprêt M, 2013, Eur Urol, v63, p1059	256	European Urology
9	Soria F, 2017, World J Urol, v35, p379	227	World Journal of Urology
10	Birtle A, 2020, Lancet, v395, p1268,	224	The Lancet

Reference burst refers to the large number of citations of literature in a certain period, reflecting the significant increase in the attention and influence of this research in the academic community.^[25]^ As shown in Figure 7B, our scientometric analysis detected 10 references with significant citation surges. The graphical representation uses chromatic coding, with saturated red hues denoting years of peak citation intensity. Among these, the literature by Rouprêt M et al, “European Association of Urology Guidelines on Upper Urinary Tract Urothelial Carcinoma: 2020 Update^[26]^” exhibited the largest citation burst from 2021 to 2025, with an intensity of 139.08. Another reference, also published by Rouprêt M et al, “European Association of Urology Guidelines on Upper Urinary Tract Urothelial Carcinoma: 2017 Update^[24]^” underwent a significant citation burst from 2019 to 2021, with an intensity of 127.75, representing the second-largest impact burst. Overall, the citation burst intensities of these 10 references ranged from 43.00 to 139.08, and the duration of their influence spanned between 2 to 4 years.

## 4. Discussion

### 4.1. General information

This investigation represents the inaugural comprehensive bibliometric evaluation of UTUC, systematically examining both contemporary research patterns and prospective developmental trajectories in this field. Our bibliometric analysis, drawing upon WoSCC data current through February 1, 2025, identified a corpus of 3621 UTUC-related articles published in 496 journals, representing the work of 14,918 authors from 3409 institutions spanning 83 countries. Through visualization analysis using CiteSpace and VOSviewer software, we systematically investigated geographical and temporal publication trends, author contribution metrics and journal dissemination patterns. Through the literature method and keyword co-occurrence analysis, we identified the research knowledge base, hotspots, frontiers, and defined the core evolution path of the theme in each period. Then, we determined the current research frontier of UTUC.

From 2000 to 2006, the number of published articles globally related to UTUC was <50, indicating that the field was in its infancy stage with inadequate foundations. Subsequently, from 2007 to 2017, there was a moderate increase in the number of published papers. However, from 2018 to 2024, there was a significant surge in research output, with an overall trend of rapid development, averaging 200 papers published per year. This suggests that the field related to UTUC remains a prominent area of research.

Bibliometric data identifies 3 primary contributing nations: the United States (n = 1207), Japan (n = 649), and China (n = 635). Examination of institutional affiliations indicates that 80% of top-performing research centers are located in either Western Europe or East Asia. Although strong transnational partnerships have developed among North American and Central European countries, our analysis reveals insufficient cooperation intensity between institutions worldwide. A particularly striking example is the minimal collaborative output between Taiwanese and Chinese research entities. To foster scientific breakthroughs, we recommend establishing multilateral cooperative frameworks to strengthen global UTUC research networks.

Through systematic examination of periodical attributes and citation networks, scholars can obtain critical data to inform their decisions when selecting target journals for manuscript submission v. As Table 5 indicates, “UROL ONCOL-SEMIN ORI” (n = 193, IF2.40) is the most prominent journal in this field, publishing the highest number of papers. Meanwhile, the journal with the most co-citations is the “Journal of Urology.” These 2 journals share numerous research directions in cancer treatment, cancer drug resistance and prognostic studies, particularly in the diagnosis and treatment strategies for urinary system tumors. The impact factor analysis reveals Journal of Clinical Oncology (IF = 42.1) as the foremost publication, followed by European Urology (IF = 25.3). Co-citation evaluation demonstrates that influential Q2 journals constitute a major portion of frequently cited references in this domain. As depicted in Figure 5, the discipline-based journal mapping delineates 2 key citation pathways: molecular/biological sciences to clinical medicine and health/nursing research to clinical medicine, illustrating the evolution of UTUC research from basic science to clinical implementation.

From the author’s perspective, Shariat, Shahrokh F, Roupret, Morgan, and Margulis, Vitaly stand out as exceptional and prolific contributors. Shariat, Shahrokh F leads the pack with the highest number of published papers at 221, followed by Roupret, Morgan with 143 papers and Margulis, Vitaly with 114. Among the 6 authors who have been cited >600 times, Rouprêt, M. tops the list with 2101 citations, closely followed by Margulis, V. with 974, Seisen, T. with 661, and Lughezzani, G. with 649. Professor Shariat, Shahrokh F, in his extensive research career spanning 221 papers, has delved deeply into the diagnosis, risk stratification, treatment, and prognostic factors of UTUC. His work also includes investigations into the recurrence mechanisms of non-muscle-invasive bladder cancer, offering clinical insights into managing and preventing the recurrence of this disease.

Rouprêt, M has the highest co-citation frequency and is one of the primary authors of the UTUC section in the “European Association of Urology Guidelines.” His involvement in the compilation of “European Association of Urology Guidelines on Upper Urinary Tract Urothelial Carcinoma: 2017 Update^[24]^” has had a significant impact on the field. This guideline provides a systematic approach to the diagnosis, staging, treatment, and follow-up of UTUC, which is widely used in clinical practice. It also offers a standardized treatment protocol for urologists worldwide, significantly influencing the clinical management of UTUC. Additionally, his other article “Erdafitinib in BCG-treated high-risk non-muscle-invasive bladder cancer,^[27]^” the efficacy of the FGFR inhibitor Erdafitinib was investigated in high-risk non-muscle-invasive bladder cancer patients who experienced BCG treatment failure. The results demonstrated that among patients with FGFR genetic alterations, the complete response rate reached 93%, and the median duration of response was not reached. This enabled all responders to avoid cystectomy within 12 months, indicating that Erdafitinib represents a highly promising targeted therapy for this patient population.

### 4.2. Hotspots and frontiers

#### 4.2.1. Diagnosis of UTUC

The clinical symptoms of UTUC are often insidious and may be difficult to detect in the early stages. Common clinical manifestations include painless hematuria, lumbar pain, urinary frequency, urinary urgency, and dysuria. Therefore, its diagnosis typically requires multidisciplinary collaboration. In terms of imaging examination, CT is a commonly used diagnostic tool for UTUC.^[28]^ Specifically, CT urography, with its high resolution, rapid imaging capability and comprehensive evaluation of the urinary system, enables precise tumor localization and determination of the extent of invasion.^[29]^ The mechanism involves the injection of contrast agents to enhance urinary tract imaging, while CT captures cross-sectional images of the kidneys and urinary tract from multiple angles. Furthermore, for patients with renal insufficiency or contrast agent allergies, magnetic resonance imaging serves as a suitable alternative. Magnetic resonance imaging provides high-resolution soft tissue images and is particularly valuable in assessing local tumor invasion, the relationship between the tumor and surrounding structures and lymph node metastasis.^[30]^ Secondly, URS is the gold standard for diagnosing UTUC.^[31]^ This technique allows direct access to the urinary system, enabling physicians to visually inspect the presence, size, morphology, and location of tumors, as well as their relationship with adjacent tissues. Compared to imaging examinations, URS provides more detailed visual images and allows for more precise determination of tumor location and extent. Its high accuracy, minimally invasive nature, integration of diagnosis and treatment and visualization capabilities make URS an indispensable tool in the management of UTUC. Finally, urine tests can serve as auxiliary diagnostic methods. Although their sensitivity and specificity are lower than those of imaging or endoscopic examinations, they still play an important role in the diagnosis and follow-up of UTUC. Despite the advantages of being noninvasive and highly specific, urine tests have limited detection capability for low-grade tumors due to their low sensitivity, and they may yield false-positive or false-negative results.^[32,33]^ Therefore, urine tests cannot fully replace imaging and endoscopic examinations. In recent years, As artificial intelligence (AI) technology has emerged as a prominent research focus, its applications in areas such as imaging diagnosis, pathological slide analysis, and prognosis prediction have provided novel tools and methodologies for the precise diagnosis and treatment of diseases.^[34,35]^ Future research is expected to deeply integrate AI technology into the imaging diagnosis, pathological analysis, and prognosis prediction of UTUC. In terms of disease diagnosis, AI can analyze digital pathology and whole slide images through deep learning to identify tumor features that are difficult to recognize with the naked eye. This enables accurate prediction of postoperative survival outcomes and responses to adjuvant chemotherapy, providing critical evidence for personalized treatment.^[36,37]^ While traditional diagnostic methods remain foundational, the integration of AI holds promise for enhancing diagnostic accuracy and prognostic prediction in UTUC.

#### 4.2.2. Immunotherapy and molecular targeted therapy for UTUC

In recent years, immunotherapy and molecular targeted therapy for UTUC have become research hotspots.^[38,39]^ These therapeutic modalities primarily link tumor molecular characteristics with individual biological features to formulate personalized treatment plans. Central to this therapeutic approach is the FGFR signaling pathway, which comprises 4 receptor subtypes (FGFR1–FGFR4). Pathogenic alterations in FGFR genes are commonly observed in various malignancies, including lung cancer, breast cancer, endometrial malignancies, and cutaneous squamous cell carcinoma.^[40]^

**Figure 8.**
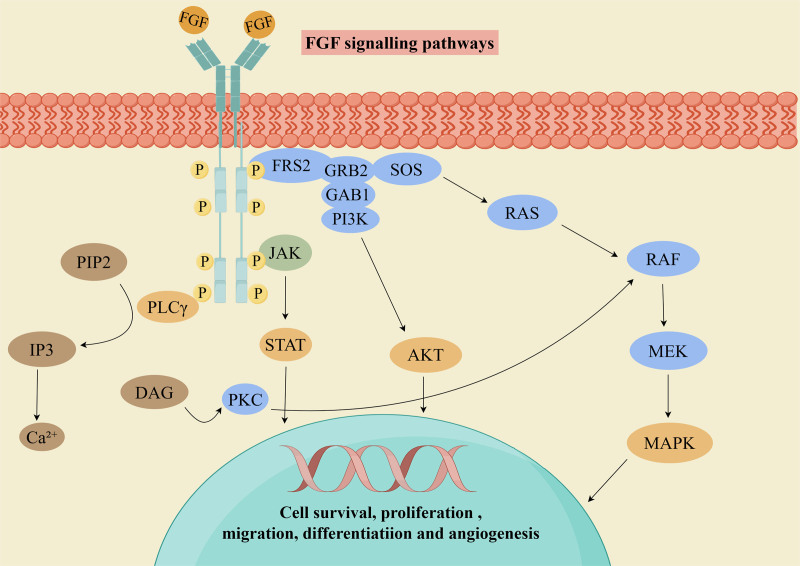
FGFR signaling pathway diagram. FGFR = fibroblast growth factor receptors.

The FGFR-mediated signal transduction pathways include RAS–RAF–mitogen-activated protein kinase (MAPK), phosphatidylinositol 3-kinase–protein kinase B (PI3K–AKT), signal transducer and activator of transcription, and phospholipase Cγ, all of which are essential for the growth and differentiation of normal cells.^[41]^ When FGFR is mutated, it leads to overactivation of the RAS–RAF–MAPK pathway, which stimulates cell proliferation and differentiation. In contrast, hyperactivation of the PI3K–AKT pathway inhibits apoptosis (Fig. 8). The signal transducer and activator of transcription pathway is associated with the promotion of tumor invasion, metastasis and enhanced immune evasion capacity.^[42]^ The phospholipase Cγ signaling pathway serves as a key regulator of tumor cell metastasis. Among these, FGFR3 gene mutation is one of the common genetic alterations in UTUC, leading to constitutive receptor activation and thereby promoting tumor growth. FGFR3 mutations are often indicative of a more favorable prognosis and lower malignancy grade, suggesting reduced invasiveness and a lower risk of recurrence and progression.^[43]^ Therefore, this target represents a potential therapeutic direction.^[44]^

Currently, FGFR inhibitors have become a hotspot in targeted drug development. The representative drug erdafitinib has demonstrated potential in clinical trials. As an oral FGFR kinase inhibitor, it selectively inhibits the activity of FGFR1-4, blocking downstream signaling pathways such as the MAPK and PI3K/AKT pathways, thereby suppressing tumor cell proliferation and progression.^[45]^ The development of FGFR inhibitors offers new hope for targeted therapy in patients with FGFR-driven tumors.

In contrast to FGFR3, the impact of mutations in another gene, TP53, is more complex. The mutation rate of TP53 is relatively low in UTUC and is more frequently observed in high-grade tumors.^[46]^ These mutations are generally associated with a more aggressive tumor phenotype, higher metastatic potential and poorer prognosis.^[47]^ TP53 mutations often coexist with mutations in other genes, leading to increased genome instability in tumor cells. Such instability may promote the development of tumor heterogeneity, thereby contributing to the emergence of drug resistance. Currently, researchers are exploring small-molecule drugs capable of restoring p53 function in the context of TP53 mutations.^[48]^ Among these, the most representative agent is Rezatapopt (PC14586), which functions as a “molecular glue” to precisely bind and correct the Y220C-specific hotspot mutation in TP53, thereby restoring its tumor suppressor activity. This drug is currently being utilized in the treatment of various advanced solid tumors.^[49]^

In UTUC, sustained activation of the PI3K–AKT signaling pathway is a key factor contributing to resistance against cisplatin-based chemotherapy and immunotherapy, which significantly increases the difficulty of treatment. Currently, breakthroughs have been achieved in the development of targeted agents against this pathway. For instance, PI3K inhibitors can block PI3K–AKT signaling,^[50]^ AKT inhibitors function by directly suppressing AKT activity,^[51]^ and mTOR inhibitors can impede protein synthesis to delay tumor progression. Meanwhile, immunotherapies such as PD-1/PD-L1 inhibitors (e.g., nivolumab) have also emerged as significant advances in the treatment of cancer, including UTUC.^[52]^

#### 4.2.3. Advances in surgical and endoscopic techniques for UTUC

Significant progress has been made in recent years in the surgical and endoscopic techniques for UTUC, driven by the advancements in precision medicine. UTUC often occurs in the renal pelvis or ureter, and due to its complex anatomical location, high recurrence rate and potential for metastasis, surgical treatment can be challenging. For surgical options, RNU and nephron-sparing surgery (NSS) are the 2 primary choices. Firstly, RNU serves as the standard treatment for UTUC, suitable for most high-risk UTUC patients, especially those with muscle-invasive (≥T2 stage) or high-risk non-muscle-invasive UTUC, by completely removing the kidney and ureter, it minimizes the risk of tumor recurrence.^[53]^ For UTUC patients with widespread tumors or deep-seated tumors, RNU ensures extensive resection of cancerous tissues, providing a more direct and thorough treatment effect. However, RNU involves the removal of the kidney and ureter, potentially leading to kidney loss for the patient. This is particularly significant for patients with a solitary kidney, as the loss of a healthy kidney can render it nonfunctional, possibly resulting in end-stage renal disease and the need for long-term dialysis. Additionally, RNU is a major surgical procedure, inherently increasing surgical trauma and prolonging the patient’s recovery period, thereby elevating the risk of complications such as DNA damage, infection and poor wound healing. In recent years, NSS has gradually emerged as a renal-preserving surgical option. It is suitable for patients with UTUC who have localized lesions or meet the criteria for surgical renal preservation. NSS involves the removal of the damaged site (typically tumors in the ureter or renal pelvis) while preserving renal function, thereby avoiding extensive nephron resection.^[54]^ Its primary advantage lies in the ability to maintain patients’ renal function, which minimizes the negative impact on their quality of life. This surgical approach is particularly crucial for high-risk patients, such as those with renal insufficiency or solitary kidneys. Compared to RNU, NSS typically involves a smaller surgical resection scope. Consequently, patients experience a faster recovery time, enabling them to return to normal life and work more quickly. Additionally, since nephrons are preserved, patients do not require dialysis or other treatments, thereby reducing health risks.

It is noteworthy that in recent years, ureterorenoscopy has gradually emerged as a significant technique for treating UTUC. This approach is particularly suitable for localized lesions and for patients who are unsuitable for open surgery. Through the ureteroscope, doctors can directly resect or cauterize the tumor site, resulting in smaller wounds and faster recovery. Its high-frequency electrocautery and laser cutting techniques enable precise tumor resection with minimal damage to normal tissue. Advancements in endoscopic technology, including the introduction of high-resolution endoscopes, advanced laser systems, and simpler operating tools, have made endoscopic treatment more precise and convenient.^[55]^ These advancements not only provide high-definition images but also allow precise lesion localization and resection. Future research progress may involve the use of minimally invasive surgery, robot-assisted surgery, AI technology to aid in precise diagnosis and surgical resection, as well as combined therapies such as surgery and blockade therapy to improve patient survival rates and quality of life.

Despite numerous advancements in surgical and endoscopic techniques for UTUC treatment, several challenges remain. For instance, due to the high recurrence and metastasis risk of UTUC, many patients still require further chemotherapy or immunotherapy to reduce mutation risks even after surgical treatment. Additionally, balancing tumor resection and renal function preservation remains a technical challenge in NSS. Therefore, further high-quality research is needed to confirm surgical approaches, long-term safety, recurrence and metastasis of UTUC for future clinical applications.

### 4.3. An overview of key clinical trials for UTUC treatment

We systematically summarized the key clinical studies, providing an integrated resource for the diversification of UTUC treatment strategies, these key clinical trials are systematically summarized in Table 8.^[56–65]^ The trends demonstrated by these trials indicate a synergistic shift in clinical practice toward integrating nephron-sparing approaches with biomarker-driven therapies. Key studies include: The POUT trial (NCT01993979), which confirmed that adjuvant gemcitabine–platinum chemotherapy after nephroureterectomy improves disease-free survival.^[56]^ The OLYMPUS trial (NCT02793128), which demonstrated favorable efficacy of primary chemoablation with UGN-101 in low-grade UTUC.^[57]^ The PROOF 302 trial (NCT04197986), which was terminated early but highlighted the clinical challenges of targeting FGFR3 alterations with infigratinib.^[58]^ A multicenter phase II trial of neoadjuvant gemcitabine plus cisplatin (NCT01261728), which showed significant pathological responses.^[59]^ A phase I trial of WST-11 vascular-targeted photodynamic therapy (NCT03617003), offering a safe option for renal function preservation.^[60]^ A prospective trial of extraperitoneal laparoscopic extended retroperitoneal lymph node dissection (NCT03544437), confirming the feasibility of the procedure and its role in reducing recurrence risk.^[61]^ A phase II study of neoadjuvant tislelizumab (NCT04672330), highlighting its efficacy in cisplatin-ineligible patients.^[62]^ The final report of the OLYMPUS trial (NCT02793128) on long-term outcomes of UGN-101, affirming clinically meaningful response rates despite potential adverse events.^[63]^ A multicenter retrospective comparative analysis of neoadjuvant versus adjuvant chemotherapy, suggesting superior outcomes with neoadjuvant therapy in high-risk cases,^[64]^ and a phase II trial of accelerated neoadjuvant chemotherapy with methotrexate, vinblastine, doxorubicin, and cisplatin, which achieved notable rates of pathological complete response collectively.^[65]^ It is noteworthy that 2 distinct references^[57,63]^ utilized data from the same clinical trial (NCT02793128), but correspond to publications from different phases or with different analytical focuses. This is a common practice in academic papers and clinical trial reports, particularly in large multi-phase clinical trials. These studies untangle the ongoing transition toward personalized and minimally invasive management in UTUC, thereby guiding future research directions.

**Table 8:** Clinical trials focusing on UTUC.

Number	Clinical trial registration number	Study title	References	Intervention	States	Conclusion
1	NCT01993979	Adjuvant chemotherapy in upper tract urothelial carcinoma (the POUT trial): a phase 3, open-label, randomized controlled trial	DOI: 10.1016/S0140-6736(20)30415-3.^[56]^	Cisplatin + carboplatin + gemcitabine	Unknown	Gemcitabine-platinum combination chemotherapy initiated within 90 days after nephroureterectomy significantly improved disease-free survival in patients with locally advanced UTUC.
2	NCT02793128	Primary chemoablation of low-grade upper tract urothelial carcinoma using UGN-101, a mitomycin-containing reverse thermal gel (OLYMPUS): an open-label, single-arm, phase 3 trial	DOI: 10.1016/S1470-2045(20)30147-9.^[57]^	UGN-101 instillations	Completed	Primary chemoablation of low-grade upper tract urothelial cancer with intracavitary UGN-101 results in clinically significant disease eradication and might offer a kidney-sparing treatment alternative for these patients.
3	NCT04197986	Targeting FGFR3 alterations with adjuvant infigratinib in invasive urothelial carcinoma: the phase III PROOF 302 trial	DOI: 10.2217/fon-2021-1629.^[58]^	Infigratinib + placebo	Terminated	The study failed to demonstrate clinical benefits of infigratinib in patients with invasive urothelial carcinoma (UC) harboring FGFR3 alterations, highlighting the necessity for further research.
4	NCT01261728	Multicenter phase II clinical trial of gemcitabine and cisplatin as neoadjuvant chemotherapy for patients with high-grade upper tract urothelial carcinoma	DOI: 10.1200/JCO.22.00763.^[59]^	Gemcitabine + cisplatin	Ongoing	NAC with split-dose GC for high-risk UTUC is a well-tolerated, effective therapy demonstrating evidence of pathologic response that is associated with favorable survival outcomes.
5	NCT03617003	Final results of a phase I trial of WST-11 (TOOKAD soluble) vascular-targeted photodynamic therapy for upper tract urothelial carcinoma	DOI: 10.1097/JU.0000000000003202.^[60]^	WST11 mediated vascular targeted phototherapy (VTP)	Ongoing	Vascular-targeted photodynamic therapy with WST-11 has an acceptable safety profile with strong potential as an effective, kidney-sparing endoscopic management option for upper tract urothelial carcinoma.
6	NCT03544437	Prospective clinical trial of the oncologic outcomes and safety of extraperitoneal laparoscopic extended retroperitoneal lymph node dissection at time of nephroureterectomy for upper tract urothelial carcinoma	DOI: 10.3389/fonc.2022.791140.^[61]^	Extraperitoneal laparoscopic extended retroperitoneal lymph node dissection	Completed	extraperitoneal laparoscopic extended LND during extraperitoneal laparoscopic RNU for UTUC is a feasible and safe procedure, which provides apotentially lower risk of regional LN recurrence.
7	NCT04672330	A phase 2 study of tislelizumab as neoadjuvant treatment of cisplatin-ineligible high-risk upper tract urothelial carcinoma	DOI: 10.1097/JU.0000000000004475.^[62]^	Tislelizumab	Completed	Neoadjuvant tislelizumab showed promising efficacy and manageable toxicity in high-risk, cisplatin-ineligible UTUC patients.
8	NCT02793128	Durability of response to primary chemoablation of low-grade upper tract urothelial carcinoma using UGN-101, a mitomycin-containing reverse thermal gel: OLYMPUS trial final report	DOI: 10.1097/JU.0000000000002350.^[63]^	UGN-101 instillations	Completed	UGN-101 provides an effective renal preservation treatment option for patients with low-grade UTUC, and its complete remission rate and remission durability are clinically meaningful. However, maintenance therapy may increase the risk of adverse events such as ureteral stricture and the benefits need to be weighed against the risks in clinical application.
9		Comparison of neoadjuvant and adjuvant chemotherapy for upper tract urothelial carcinoma in real-world practice: a multicenter retrospective study	DOI: 10.1093/jjco/hyad118.^[64]^	Neoadjuvant chemotherapy + adjuvant chemotherapy	Completed	Neoadjuvant chemotherapy would be more effective in high-risk upper tract urothelial carcinoma patients compared with adjuvant chemotherapy. However, no clinical trial has established the superiority of neoadjuvant chemotherapy or adjuvant chemotherapy in terms of perioperative outcomes.
10		Phase II trial of neoadjuvant systemic chemotherapy followed by extirpative surgery in patients with high grade upper tract urothelial carcinoma	DOI: 10.1097/JU.0000000000000644.^[65]^	Methotrexate + vinblastine + doxorubicin + cisplatin	Completed	Accelerated methotrexate, vinblastine, doxorubicin and cisplatin neoadjuvant chemotherapy in patients with high grade upper tract urothelial carcinoma and creatinine clearance >50 ml per minute was safe and demonstrated predefined activity with a 14% pathological complete response rate. Final pathological stage ypT1 or less in more than 60% of patients

UTUC = upper tract urothelial carcinoma.

## 5. Strengths and limitations

This bibliometric analysis provides a comprehensive overview of UTUC research; however, several methodological limitations warrant acknowledgments. First, the keyword strategy, although designed to enhance topic specificity, may inadvertently exclude conceptually relevant studies, potentially leading to omissions within the broader UTUC field. Second, reliance solely on the WoSCC database, while beneficial for standardization, may underestimate applied or interdisciplinary research that is more extensively indexed in databases such as PubMed or Scopus. Third, citation-based metrics are inherently time-sensitive and tend to disadvantage recently published studies, reflecting academic rather than translational impact. Fourth, the exclusion of non-English publications to ensure language consistency may introduce geographical bias, potentially overlooking research contributions from non-English-speaking countries. Finally, a certain degree of subjectivity is unavoidable during the interpretation and synthesis of bibliometric analysis results.

## 6. Conclusion

Through visualization analysis of UTUC publications over a 25-year timeframe (2000–2025), we identify sustained growth in research productivity, led principally by American and Japanese scholars. Key areas of scientific focus include mutational profiles (FGFR3/TP53), precision oncology strategies and immunological mechanisms, indicating promising directions for therapeutic development. While anticipation builds for innovative treatment paradigms, the discipline faces a pressing need for higher-level evidence through well-designed clinical trials and meta-analyses to translate basic science findings into clinical practice. Future research should focus on validating biomarker-driven therapies and expanding international collaborative networks to enhance the translational impact of UTUC studies. This comprehensive assessment offers valuable insights into the structural and temporal evolution of UTUC research.

## Author contributions

**Conceptualization:** Xiaohan Ma, Xin Tang, Yan Zhang, Encun Hou.

**Data curation:** Xiaohan Ma, Tong Wu, Xin Tang, Yan Zhang, Encun Hou.

**Formal analysis:** Shuying Wang, Sheng Chen, Xiaohan Ma, Tong Wu, Xin Tang, Yan Zhang, Encun Hou.

**Funding acquisition:** Shuying Wang, Sheng Chen, Xiaohan Ma, Tong Wu, Xin Tang, Yan Zhang, Xiaofei Zhang, Encun Hou.

**Investigation:** Shuying Wang, Sheng Chen, Xiaohan Ma, Tong Wu, Xin Tang, Yan Zhang, Encun Hou.

**Methodology:** Shuying Wang, Sheng Chen, Xiaohan Ma, Tong Wu, Peiling Zuo, Xin Tang, Yan Zhang, Xiaofei Zhang, Encun Hou.

**Project administration:** Shuying Wang, Sheng Chen, Tong Wu, Peiling Zuo, Xiaofei Zhang.

**Resources:** Shuying Wang, Sheng Chen, Peiling Zuo.

**Software:** Peiling Zuo, Xiaofei Zhang.

**Supervision:** Peiling Zuo, Xiaofei Zhang.

**Validation:** Peiling Zuo.

**Visualization:** Xiaofei Zhang.

## References

[1] Alessandra C Anna C Riccardo V. MicroRNA signatures in the upper urinary tract urothelial carcinoma scenario: ready for the game changer? Int J Mol Sci. 2022;23:158–67. 10.3390/ijms23052602 PMC8910117 35269744

[2] Deng L Yun H Zesong Y Liefu Y. Therapeutic strategies for asymptomatic upper urinary tract urothelial carcinoma. Wideochir Inne Tech Maloinwazyjne. 2023;18:343–50. 37680741 10.5114/wiitm.2022.123307 PMC10481439

[3] Weipu M Jianping W Keyi W Bin X Ming C. Marital status does not affect the cancer-specific survival of patients with upper tract urothelial carcinoma treated with nephroureterectomy: a propensity score matching study. Ther Adv Urol. 2021;12:1756287220981510. 10.1177/1756287220981510 PMC7768858 33488776

[4] Jie W Pei-Hang X Wen-Jie L. Intravesical recurrence after radical nephroureterectomy of upper urinary tract urothelial carcinoma: a large population-based investigation of clinicopathologic characteristics and survival outcomes. Front Surg. 2021;8:590448. 33693025 10.3389/fsurg.2021.590448 PMC7938894

[5] Chenchen F Lujia W Guanxiong D. Predictive value of clinicopathological markers for the metachronous bladder cancer and prognosis of upper tract urothelial carcinoma. Sci Rep. 2014;4:4015. 24500328 10.1038/srep04015 PMC3915316

[6] Li Q Chen T Zhu A. Risk factors of renal function deterioration after radical nephroureterectomy for upper tract urothelial carcinoma. Front Oncol. 2024;14:1438835. 39479018 10.3389/fonc.2024.1438835 PMC11521784

[7] Sfakianos JP Cha EK Iyer G. Genomic characterization of upper tract urothelial carcinoma. Eur Urol. 2015;68:970–7. 26278805 10.1016/j.eururo.2015.07.039 PMC4675454

[8] Seisen T Granger B Colin P. A systematic review and meta-analysis of clinicopathologic factors linked to intravesical recurrence after radical nephroureterectomy to treat upper tract urothelial carcinoma. Eur Urol. 2015;67:1122–33. 25488681 10.1016/j.eururo.2014.11.035

[9] Margulis V Shariat SF Matin SF. Outcomes of radical nephroureterectomy: a series from the Upper Tract Urothelial Carcinoma Collaboration. Cancer. 2009;115:1224–33. 19156917 10.1002/cncr.24135

[10] Mandalapu RS Matin SF. Contemporary evaluation and management of upper tract urothelial cancer. Urology. 2016;94:17–23. 26850816 10.1016/j.urology.2015.12.035

[11] Loriot Y Necchi A Park SH. Erdafitinib in locally advanced or metastatic urothelial carcinoma. N Engl J Med. 2019;381:338–48. 31340094 10.1056/NEJMoa1817323

[12] Balar AV Galsky MD Rosenberg JE. Atezolizumab as first-line treatment in cisplatin-ineligible patients with locally advanced and metastatic urothelial carcinoma: a single-arm, multicentre, phase 2 trial. Lancet. 2017;389:67–76. 27939400 10.1016/S0140-6736(16)32455-2 PMC5568632

[13] Powles T Rosenberg JE Sonpavde GP. Enfortumab vedotin in previously treated advanced urothelial carcinoma. N Engl J Med. 2021;384:1125–35. 33577729 10.1056/NEJMoa2035807 PMC8450892

[14] Ellegaard O Wallin JA. The bibliometric analysis of scholarly production: how great is the impact? Scientometrics. 2015;105:1809–31. 26594073 10.1007/s11192-015-1645-z PMC4643120

[15] Sun J Bai S Zhao J. Mapping knowledge structure and research of the biologic treatment of asthma: a bibliometric study. Front Immunol. 2023;14:1034755. 36845128 10.3389/fimmu.2023.1034755 PMC9947831

[16] Xiangnv M Zhongting L Fu M Sha S Tao L. Research hotspots and emerging trends in targeted therapy for colorectal cancer: a bibliometric analysis (2000–2023). Discov Oncol. 2025;16:789. 40380023 10.1007/s12672-025-02632-x PMC12084209

[17] Chen C. Searching for intellectual turning points: progressive knowledge domain visualization. Proc Natl Acad Sci USA. 2004;101(Suppl 1):5303–10. 14724295 10.1073/pnas.0307513100 PMC387312

[18] van Eck NJ Waltman L. Software survey: VOSviewer, a computer program for bibliometric mapping. Scientometrics. 2010;84:523–38. 20585380 10.1007/s11192-009-0146-3 PMC2883932

[19] Fan Y Xiao H Wang Y Wang S Sun H. Global research on nanomaterials for liver cancer from 2004 to 2023: a bibliometric and visual analysis. Discover Oncol. 2024;15:838. 10.1007/s12672-024-01735-1 PMC11669646 39722094

[20] Wu F Gao J Kang J. Knowledge mapping of exosomes in autoimmune diseases: a bibliometric analysis (2002–2021). Front Immunol. 2022;13:939433. 35935932 10.3389/fimmu.2022.939433 PMC9353180

[21] Long Z Jiao Z Junfeng W. Global trends and research hotspots in autophagy and tumor drug resistance: a bibliometric analysis. Discov Oncol. 2025;16:734. 40354002 10.1007/s12672-025-02379-5 PMC12069191

[22] Hang Y Tao Z Dongyang W. A systematic bibliometric analysis of cardiovascular disease risk in obesity (2014–2024). J Multidiscip Healthc. 2025;18:3233–55. 40502827 10.2147/JMDH.S504022 PMC12153957

[23] Chaoqun W Ming L Wei W. The rising influence of lipid metabolism in lung cancer: a global research perspective. Front Oncol. 2025;15:1562621. 40231255 10.3389/fonc.2025.1562621 PMC11995272

[24] Rouprêt M Babjuk M Compérat E. European association of urology guidelines on upper urinary tract urothelial carcinoma: 2017 update. Eur Urol. 2018;73:111–22. 28867446 10.1016/j.eururo.2017.07.036

[25] Yan-Jun C Ming-Rong X Sheng-Qiang Z Fang L. Synapses-associated research in Parkinson’s disease: an explored trends analysis. Front Aging Neurosci. 2025;17:1563142. 40212565 10.3389/fnagi.2025.1563142 PMC11983582

[26] Rouprêt M Babjuk M Burger M. European association of urology guidelines on upper urinary tract urothelial carcinoma: 2020 update. Eur Urol. 2021;79:62–79. 32593530 10.1016/j.eururo.2020.05.042

[27] Catto JWF Tran B Rouprêt M. Erdafitinib in BCG-treated high-risk non-muscle-invasive bladder cancer. Ann Oncol. 2024;35:98–106. 37871701 10.1016/j.annonc.2023.09.3116

[28] Chih-Ming L Jen-Jie L Han-Hsiang H. A panel of tumor markers, calreticulin, annexin A2, and annexin A3 in upper tract urothelial carcinoma identified by proteomic and immunological analysis. BMC Cancer. 2014;14:363. 24884814 10.1186/1471-2407-14-363 PMC4039341

[29] Ji Kang Y Si Hyun K Woong Bin K Hee Kyung K Sang Wook L. Simultaneous robot-assisted approach in a super-elderly patient with urothelial carcinoma and synchronous contralateral renal cell carcinoma: a case report. World J Clin Cases. 2022;10:7153–62. 36051108 10.12998/wjcc.v10.i20.7153 PMC9297406

[30] Saenthaveesuk P Yang L Zeng B. Development and validation of multiparametric MRI-based nomogram for predicting occult metastasis risk in early tongue squamous cell carcinoma. BMC Cancer. 2021;21:408. 33858377 10.1186/s12885-021-08135-6 PMC8048044

[31] Lazo JF Marzullo A Moccia S. Using spatial-temporal ensembles of convolutional neural networks for lumen segmentation in ureteroscopy. Int J Comput Assist Radiol Surg. 2021;16:915–22. 33909264 10.1007/s11548-021-02376-3 PMC8166718

[32] Dal Moro F Valotto C Guttilla A Zattoni F. Urinary markers in the everyday diagnosis of bladder cancer. Urologia. 2013;80:265–75. 24419920 10.5301/urologia.5000041

[33] Hadland SE Levy S. Objective testing: urine and other drug tests. Child Adolesc Psychiatr Clin N Am. 2016;25:549–65. 27338974 10.1016/j.chc.2016.02.005 PMC4920965

[34] Chunyu L Ming D Xiaoli Z. Multi-view radiomics and deep learning modeling for prostate cancer detection based on multi-parametric MRI. Front Oncol. 2023;13:1198899. 37448515 10.3389/fonc.2023.1198899 PMC10338012

[35] Na L Tianqing W Guohu H Minbin C. Exosome-mediated ferroptosis in the tumor microenvironment: from molecular mechanisms to clinical application. Cell Death Discov. 2025;11:221. 40328736 10.1038/s41420-025-02484-y PMC12056189

[36] Yong R Wenqi X Jiayun W. Artificial intelligence-based prediction of organ involvement in Sjogren’s syndrome using labial gland biopsy whole-slide images. Clin Rheumatol. 2025;44:2919–27. 40471393 10.1007/s10067-025-07518-5 PMC12234637

[37] Parikshit S Dipanwita B Suvradeep M. Artificial intelligence in liver pathology: precision histology for accurate diagnoses. J Clin Exp Hepatol. 2025;15:103145. 40927758 10.1016/j.jceh.2025.103145 PMC12414889

[38] Ping-Yu Z Li W Kun-Peng L Shan Y Xiao-Bin C. Perioperative and oncologic outcomes of transperitoneal versus retroperitoneal laparoscopic nephroureterectomy for upper urinary tract urothelial carcinoma: a systematic review and pooled analysis of comparative outcomes. World J Surg Oncol. 2023;21:163. 37248555 10.1186/s12957-023-03046-1 PMC10226240

[39] Ruopeng S Zeyu C Hailong H. Clinical outcomes of immune checkpoint inhibitor plus nab-paclitaxel in metastatic upper tract urothelial carcinoma. Transl Androl Urol. 2023;12:1416–25. 37814696 10.21037/tau-23-404 PMC10560336

[40] Yue H Wang Y Zhang N. Small-molecule FGFR-targeted medicinal chemistry: advances since 2020 and future perspectives. Eur J Med Chem. 2025;300:118117. 40945321 10.1016/j.ejmech.2025.118117

[41] Luyao S Yongsheng L Huakan Z. Fibroblast growth factor signaling in macrophage polarization: impact on health and diseases. Front Immunol. 2024;15:1390453. 38962005 10.3389/fimmu.2024.1390453 PMC11219802

[42] Du S Zhang Y Xu J. Current progress in cancer treatment by targeting FGFR signaling. Cancer Biol Med. 2023;20:490–9. 37493315 10.20892/j.issn.2095-3941.2023.0137 PMC10466438

[43] Crocetto F Amicuzi U Musone M. Liquid biopsy: current advancements in clinical practice for bladder cancer. J Liq Biopsy. 2025;9:100310. 40698358 10.1016/j.jlb.2025.100310 PMC12281373

[44] WenBo W Lei C GaoZhen J. Inhibition of FGFR3 upregulates MHC-I and PD-L1 via TLR3/NF-kB pathway in muscle-invasive bladder cancer. Cancer Med. 2023;12:15676–90. 37283287 10.1002/cam4.6172 PMC10417096

[45] Turner N Grose R. Fibroblast growth factor signalling: from development to cancer. Nat Rev Cancer. 2010;10:116–29. 20094046 10.1038/nrc2780

[46] Evmorfopoulos K Mitrakas L Karathanasis A Zachos I Tzortzis V Vlachostergios PJ. Upper tract urothelial carcinoma: a rare malignancy with distinct immuno-genomic features in the era of precision-based therapies. Biomedicines. 2023;11:1775. 37509415 10.3390/biomedicines11071775 PMC10376290

[47] Ugur Y Erdogan A Huseyin Aytac A. Is cystoscopy follow-up protocol safe for low-risk bladder cancer without muscle invasion? Urol Ann. 2020;12:25–30. 32015613 10.4103/UA.UA_143_18 PMC6978980

[48] Blanco-Luquin I Lázcoz P Celay J Castresana JS Encío IJ. In vitro assessment of the role of p53 on chemotherapy treatments in neuroblastoma cell lines. Pharmaceuticals (Basel). 2021;14:1184. 34832966 10.3390/ph14111184 PMC8624165

[49] Vu BT Dominique R Fahr BJ. Discovery of rezatapopt (PC14586), a first-in-class, small-molecule reactivator of p53 Y220C mutant in development. ACS Med Chem Lett. 2025;16:34–9. 39811143 10.1021/acsmedchemlett.4c00379 PMC11726359

[50] Francis L Dan Z Xin L Thomas PL. Large granular lymphocyte leukemia: from dysregulated pathways to therapeutic targets. Future Oncol. 2012;8:87–801. 22830400 10.2217/fon.12.75 PMC3464048

[51] Yiming W Xue L Fuyang S Di X Yujiong W. Vitamin D3 promotes autophagy in THP-1 cells infected with Mycobacterium tuberculosis. Exp Ther Med. 2022;23:240. 35222717 10.3892/etm.2022.11165 PMC8815057

[52] Huiguang C Xuexin X Jingxian L. Decoding tumor-fibrosis interplay: mechanisms, impact on progression, and innovative therapeutic strategies. Front Pharmacol. 2024;15:1491400. 39534084 10.3389/fphar.2024.1491400 PMC11555290

[53] Makito M Nobutaka N Katsuya A. Initial experience of complete laparoscopic radical nephroureterectomy combined with transvesical laparoscopic excision of distal ureter in patients with upper urinary tract cancer. World J Surg Oncol. 2020;18:104. 32450850 10.1186/s12957-020-01872-1 PMC7249636

[54] Tzu-Ju C Ti-Chun C Wan-Shan L. Utility of EFEMP1 in the prediction of oncologic outcomes of urothelial carcinoma. Genes (Basel). 2021;12:872. 34204134 10.3390/genes12060872 PMC8226762

[55] Marco D. Endoscopy in IBD: when and how? Diagnostics (Basel). 2023;13:3423. 37998559 10.3390/diagnostics13223423 PMC10670128

[56] Birtle A Johnson M Chester J. Adjuvant chemotherapy in upper tract urothelial carcinoma (the POUT trial): a phase 3, open-label, randomised controlled trial. Lancet (London). 2020;395:1268–77. 10.1016/S0140-6736(20)30415-3 PMC7181180 32145825

[57] Kleinmann N Matin SF Pierorazio PM. Primary chemoablation of low-grade upper tract urothelial carcinoma using UGN-101, a mitomycin-containing reverse thermal gel (OLYMPUS): an open-label, single-arm, phase 3 trial. Lancet Oncol. 2020;21:776–85. 32631491 10.1016/S1470-2045(20)30147-9

[58] Pal SK Somford DM Grivas P. Targeting FGFR3 alterations with adjuvant infigratinib in invasive urothelial carcinoma: the phase III PROOF 302 trial. Future Oncol. 2022;18:2599–614. 35608106 10.2217/fon-2021-1629

[59] Coleman JA Yip W Wong NC. Multicenter phase II clinical trial of gemcitabine and cisplatin as neoadjuvant chemotherapy for patients with high-grade upper tract urothelial carcinoma. J Clin Oncol. 2023;41:1618–25. 36603175 10.1200/JCO.22.00763 PMC10043554

[60] Yip W Sjoberg DD Nogueira LM. Final results of a phase I trial of WST-11 (TOOKAD soluble) vascular-targeted photodynamic therapy for upper tract urothelial carcinoma. J Urol. 2023;209:863–71. 36724067 10.1097/JU.0000000000003202 PMC10265489

[61] Huang J Qian H Yuan Y. Prospective clinical trial of the oncologic outcomes and safety of extraperitoneal laparoscopic extended retroperitoneal lymph node dissection at time of nephroureterectomy for upper tract urothelial carcinoma. Front Oncol. 2022;12:791140. 35280720 10.3389/fonc.2022.791140 PMC8907892

[62] Huang J Cai X Ng C. A phase 2 study of tislelizumab as neoadjuvant treatment of cisplatin-ineligible high-risk upper tract urothelial carcinoma. J Urol. 2025;213:739–52. 39999445 10.1097/JU.0000000000004475 PMC12708035

[63] Matin SF Pierorazio PM Kleinmann N. Durability of response to primary chemoablation of low-grade upper tract urothelial carcinoma using UGN-101, a mitomycin-containing reverse thermal gel: OLYMPUS trial final report. J Urol. 2022;207:779–88. 34915741 10.1097/JU.0000000000002350 PMC12721675

[64] Takahashi K Urabe F Suhara Y. Comparison of neoadjuvant and adjuvant chemotherapy for upper tract urothelial carcinoma in real-world practice: a multicenter retrospective study. Jpn J Clin Oncol. 2023;53:1208–14. 37647644 10.1093/jjco/hyad118

[65] Margulis V Puligandla M Trabulsi EJ. Phase II trial of neoadjuvant systemic chemotherapy followed by extirpative surgery in patients with high grade upper tract urothelial carcinoma. J Urol. 2020;203:690–8. 31702432 10.1097/JU.0000000000000644 PMC7735436

